# Bereaved Families’ Experiences of Treatment Withdrawal and End‐Of‐Life Care in the Intensive Care Unit: An Exploratory‐Descriptive Qualitative Study

**DOI:** 10.1111/nicc.70185

**Published:** 2025-10-25

**Authors:** Emmanuel Kwame Korsah, Shelley Schmollgruber, Alhassan Sibdow Abukari

**Affiliations:** ^1^ Division of Clinical Associates, Department of Family Medicine and Primary Care, Faculty of Health Science University of the Witwatersrand Johannesburg South Africa; ^2^ Department of Nursing Education, Faculty of Health Science University of the Witwatersrand Johannesburg South Africa; ^3^ Department of Maternal and Child Health, School of Nursing & Midwifery University of Ghana Legon, Accra Ghana

**Keywords:** bereaved family, end‐of‐life care, family‐centred care, intensive care unit, treatment withdrawal

## Abstract

**Background:**

Bereaved families may experience emotional distress, uncertainty and psychological challenges during treatment withdrawal and end‐of‐life care in the intensive care unit.

**Aim:**

To explore bereaved family members' experiences of treatment withdrawal and end‐of‐life care in the adult intensive care unit.

**Study Design:**

The study employed an exploratory descriptive qualitative design. A semi‐structured interview guide was used to gather data from seven bereaved family members. Participants were purposively sampled from an 18‐bedded adult multi‐disciplinary intensive care unit in South Africa. Interviews were transcribed verbatim, imported into qualitative software and analysed using inductive thematic analysis. This qualitative study followed the Consolidated Criteria for Reporting Qualitative Research checklist.

**Results:**

Three major themes emerged from data analysis: emotional reactions and coping, a desire for proximity and participation in care, and communication and information sharing. Families experienced shock, anxiety and emotional distress, relying on social support, faith and personal beliefs to cope with their challenges. They sought physical presence, active participation and spiritual support to cope with treatment withdrawal and end‐of‐life. Clear, compassionate communication and emotional support was critical to shaping their experience and perception of care.

**Conclusions:**

Bereaved families' experiences during treatment withdrawal in the intensive care unit are complex and multifaceted, often shaped by emotional, spiritual and communication dynamics. Most families experience grief and are inadequately prepared for the impending death of their loved one. Effective communication, family involvement, interdisciplinary collaboration and teamwork are imperative in providing quality end‐of‐life care. Healthcare providers need to recognise the patient and family as a unit of care and support them throughout the treatment withdrawal process and end‐of‐life care.

**Relevance to Clinical Practice:**

Developing and implementing programs that provide psychological support, educational resources such as informational booklets on grief, and guidance on navigating healthcare services and follow‐up care can help bereaved families cope. Similarly, adopting a holistic and family‐centred care approach that addresses the emotional, psychological and practical needs of families can improve their overall experience and satisfaction with care.

**Impact Statements**

- What is known about the topic
  ○ Bereaved families may experience emotional distress, uncertainty, and psychological challenges during treatment withdrawal and end‐of‐life care in the ICU.
  ○ Improved communication, family involvement, and bereavement support are crucial for enhancing their experiences.
  ○ Families often feel excluded from decision‐making and lack adequate preparation for the dying process, leading to long‐term emotional distress such as anxiety, depression, and complicated grief.
- What this paper adds
  ○ This qualitative study explores bereaved families' experiences with treatment withdrawal and end‐of‐life care in an adult ICU, providing in‐depth insights into their emotional response, coping mechanisms, and the perceived adequacy of communication and support from healthcare providers.
  ○ The article emphasizes the importance of a family‐centered approach to ICU end‐of‐life care, recognising the impact of emotional responses, family involvement, and communication dynamics.
  ○ The findings highlight the significance of addressing families' psychological needs through structured support systems and improving healthcare providers' communication strategies.

## 1 Introduction

End‐of‐life (EOL) care in intensive care units (ICUs) is a complicated and emotionally charged process that affects both patients and families. As intensive care settings increasingly recognise the value of patient‐ and family‐centred care, ensuring that bereaved families receive adequate support during treatment withdrawal remains an important but often overlooked aspect of ICU care. Families of critically ill patients frequently experience emotional distress, uncertainty and psychological trauma, which can have long‐term consequences such as anxiety, depression, post‐traumatic stress disorder (PTSD) and complicated grief [1, 2]. Given the emotional and psychological toll families face, there is an increasing need for research into their experiences to inform strategies for improving end‐of‐life care and bereavement support.

### 1.1 Background

Treatment withdrawal refers to the purposeful discontinuation of life‐sustaining interventions, such as mechanical ventilation or vasoactive medications, when ongoing treatment is deemed physiologically inappropriate or no longer in the patient's best interest [3]. This process is closely associated with EOL care, which encompasses the provision of comprehensive support to critically ill and dying patients and their families when curative treatment is no longer feasible, with a focus on comfort, symptom management and dignified death [4, 5]. While the withdrawal of treatment and EOL care in adult ICUs is a common practice worldwide, its frequency and implementation differ across countries [6, 7]. In South Africa, active treatment withdrawal occurs less frequently compared to the other regions [7, 8]. Existing literature suggests that, although recognised and practised, treatment withdrawal is approached with caution due to ethical, cultural and systematic factors influencing EOL decision‐making in South African ICUs [5, 8].

Effective communication during the withdrawal of life‐sustaining treatment is critical for ensuring that families understand the procedure and feel supported. However, previous research has found that communication in ICUs is frequently inadequate, leaving families feeling uninformed and unprepared for their loved one's death [9, 10, 11]. Although ICU professionals provide sub‐optimal information on EOL care to families, research indicates that families are extremely attentive to their loved ones' condition and actively seek to understand the reasoning behind treatment decisions [10]. Poor communication and insufficient emotional support can worsen the psychological impact of bereavement and impede coping mechanisms [11].

Despite a wealth of international research on family experiences in ICU and end‐of‐life care, there is still a significant gap in understanding how bereaved families experience treatment withdrawal in South African healthcare settings. In resource‐limited settings, the ICU's focus on life‐saving interventions can make the transition to EOL care challenging. Constraints, such as staffing shortages, cultural dynamics, and the limited integration of palliative care services, may hinder meaningful family involvement [12, 13]. Despite these challenges, maintaining a compassionate, family‐centred approach remains essential to ensuring dignified and quality EOL care. Furthermore, nurses play crucial roles during treatment withdrawal, providing both physical and emotional support to patients and families. Such roles involve facilitating informed EOL decision‐making, advocacy, information sharing and communication, emotional support, ensuring comfort, and advocating for a peaceful and dignified death [14, 15]. Despite these significant roles, there is often less emphasis on explaining the physical symptoms of death, leaving families unprepared [16].

In South Africa, where ICU capacity and access to palliative care services are limited, understanding how families experience treatment withdrawal and EOL care is critical for improving care delivery. Cultural and religious beliefs significantly shape how families experience and interpret treatment withdrawal and EOL care in the ICU [17, 18]. However, these deeply rooted influences are often overlooked during the EOL decision‐making process. Religious and sociocultural contexts profoundly affect how surviving family members navigate the emotional and ethical complexities of treatment withdrawal [18, 19]. For many families, religion, customs, and traditional beliefs inform their understanding of death and influence the extent to which they accept or resist the withdrawal of life‐sustaining treatment [18, 19, 20]. Cultural norms also dictate who is responsible for making such decisions, how grief is expressed, and the nature of family dialogue during this critical time [17, 18, 19]. A disconnect between medical realities and cultural expectations may result in tension, frustration or mistrust between families and healthcare providers [17].

To support families more effectively during EOL care, it is crucial to provide culturally sensitive care that acknowledges these religious and sociocultural dynamics. These practices demonstrate the need for a nuanced approach to EOL care that respects family structures and belief systems. Given these complexities, there is a pressing need for research to explore how bereaved families in ICU settings experience treatment withdrawal, their coping mechanisms, and the nature of support provided by healthcare professionals. This study seeks to fill this research gap by investigating the experiences of bereaved family members during treatment withdrawal and EOL care in a multidisciplinary ICU. Using an exploratory descriptive qualitative approach, this study aims to explore bereaved family members' experiences of treatment withdrawal and EOL care in the adult ICU. The findings will guide recommendations for improving family support, improving communication strategies, and developing holistic end‐of‐life care models that consider the needs of both patients and their families.

### 1.2 Aim of the Study

The study aims to explore bereaved families' experiences of treatment withdrawal and EOL care in the adult ICU. The research question was “How do bereaved families experience treatment withdrawal and EOL care in the ICU and how do religious and sociocultural factors shape these experiences?”

## 2 Design and Methods

### 2.1 Design

The study used an exploratory, descriptive qualitative design. The study adopted this design to explore bereaved family members' experiences of treatment withdrawal and end‐of‐life care in the adult ICU. The design was executed using semi‐structured individual interviews to engage the participants for the evidence to emerge [21]. The study conforms to the consolidated criteria for reporting qualitative research guidelines [22].

### 2.2 Setting

The study was conducted in a specialist, academic‐affiliated referral hospital in South Africa, with participant recruitment taking place in an 18‐bed multidisciplinary ICU. The setting is a high‐acuity setting that serves as a primary referral centre for the neighbouring regional hospitals and health centres in Johannesburg. The unit manages a wide range of complex adult cases, including medical, surgical and obstetric/gynaecological patients.

### 2.3 Sample and Participants

The study adopted a purposive sampling approach to identify potential family members whose loved ones had been admitted to the adult ICU and visited their relatives regularly. The participants were selected on the basis that they had a relative admitted to the ICU who had treatment withdrawn at some point during their care and died in the ICU, bereaved in the last 6 months or more, an adult relative (parent, sibling, spouse) above 18 years, fluent in English and willing to share his or her experience. Participants who had been bereaved for at least 6 months were selected to allow sufficient time for initial acute grief to subside, enabling more reflective insights and minimising emotional distress during interviews.

### 2.4 Data Collection

Data collection was conducted through semi‐structured individual interviews with bereaved family members. An interview guide (Table 1) was developed based on the study objective, and the probes were supported through literature control. This approach ensured that the data gathered addressed the main aim of the study and allowed for alignment with existing literature. The interview guide was pretested using individuals possessing similar characteristics to the study population. Data from the pretest were excluded from the final analysis to maintain the rigour of the interview guide.

**TABLE 1:** Participants interview guide.

Can you tell me about your experience with your relative's admission to the ICU? How did you feel when you first learned that your loved one was critically ill and transitioning to palliative care was necessary? Probes: – Can you describe your emotional state at that time? – How did the ICU experience affect you emotionally? – Were there particular moments that were especially difficult? How did you cope during your loved one's ICU stay? – What helped you get through that time? How important was it for you to be physically present with your loved one in the ICU? Probes – Were there any restrictions or challenges? – How did that affect you? Were you given opportunities to participate in your loved one's care? – What kind of activities were you involved in? – How did participating (or not participating) affect your experience? – Were your spiritual or religious needs met? How would you describe the communication between the ICU staff and your family? – Did you feel informed about what was happening? – Were serious discussions handled in a private and respectful manner? Reflecting on your loved one's ICU stay, is there anything you wish you could have done differently?

Participant recruitment occurred during routine daily ICU ward rounds. During these ward rounds, the researchers [SS and EKK] identified patients with poor prognoses, as indicated by clinical notes such as “not for further escalation” and “futility of care” on their ICU chart. Families of these patients were initially engaged during routine counselling sessions conducted by attending ICU physicians, where the poor prognosis and decisions regarding the limitations of care were first discussed. Following these sessions, and with the attending physician's permission, the researchers (SS and EKK) approached family members to introduce the study. Permission was consistently granted by the attending doctors.

During the first point of contact, the researcher (EKK) explained the study's purpose and procedures to the families. Informed consent was obtained from those who agreed to participate, with interviews scheduled to take place six or more months after the ICU experience. For participants who consented to participate in the study, their contact details, including a phone number, email, and residential address, were recorded for follow‐up. Therefore, the study took place six or more months following the ICU experience. The entire participant recruitment took place between October 8 and December 10, 2021. To minimise bias and maintain neutrality, all the researchers had no prior relationship with participants.

In June 2022, the researchers (SS and EKK) followed up with bereaved families via phone to further explain the study, offer support if needed, and confirm interest in participating again. The researchers (SS and EKK) followed up on 15 bereaved family members, but only 10 of them were reachable. Among those we could reach, nine consented to participate in the study after the study's purpose and procedures were explained to them. For those who agreed, a study information sheet, informed consent for the interview, and an audio recording were shared in person or via email. SS carried out the interviews. Finally, seven bereaved families agreed, signed the consent forms and were interviewed. Families living nearby agreed to be interviewed at their home or at another convenient location. Due to COVID‐19 precautions, some interviews were conducted online. Interviews took place from June to September 2022. Interview sessions lasted between 30 and 40 min. Data saturation was achieved when no new themes or subthemes emerged during the analysis. The researchers determined that saturation was reached after the seventh interview, as subsequent data reflected thematic repetition without generating any new insights.

### 2.5 Data Analysis

The three researchers used an inductive thematic analysis approach, guided by Braun and Clarke's method, to analyse the data [23]. The first author (EKK) and third author (ASA) listened to the audio recordings from interviews and transcribed them verbatim into English text. The researcher (EKK) imported the transcripts into MAXQDA to aid the qualitative data analysis process [24]. The three researchers cleaned the data prior to the data analysis. Field notes from interviews were included to enrich the analysis. The six steps followed in the study's inductive thematic analysis were familiarisation with the data, generating initial codes, searching for themes, reviewing the themes, defining and naming the themes, and producing the report [23]. A detailed description of the thematic analysis as applied in this study is presented in Table 2.

**TABLE 2:** Description of thematic analysis and how it was applied in the study.

Step of thematic analysis	Application in the study
Step 1: Familiarisation with the data	The authors (SS, EKK and ASA) independently read and re‐read the interview transcripts multiple times to gain deep familiarity with the content, noting their initial impressions and potential patterns relevant to the study aim.
Step 2: Generating initial codes	Initial codes were generated inductively across the entire data set. These codes were applied to text segments that appeared meaningful, recurrent or conceptually relevant.
Step 3: Searching for themes	The authors (SS, EKK and ASA) collated related codes into potential themes by identifying patterns of shared meaning. The authors also grouped similar coded extracts under these themes and developed subthemes where appropriate to capture nuance in participants' accounts
Step 4: Reviewing the themes	Emerging themes were reviewed and refined in relation to the coded extracts and the data set to ensure coherence and consistency. The authors (SS, EKK and ASA) engaged in iterative discussions to ensure internal coherence, resolve discrepancies and confirm that themes accurately represented the data.
Step 5: Defining and naming the themes	Each theme and subtheme was clearly defined and named to reflect its core essence. Descriptive labels were developed to convey the underlying meaning and scope of each theme.
Step 6: Producing the report	The final themes were organised into a coherent narrative and presented in the findings. Participant quotes were selected to illustrate each theme, providing rich, contextual insight and supporting the interpretations made.

### 2.6 Ethical Considerations

The research followed the principles of the Declaration of Helsinki [25]. We obtained ethics approvals from the Human Research Ethics Committee (Medical) of the University of the Witwatersrand (Protocol Number: M210229, 17 September 2021) and the Johannesburg Academic Hospital (Ethic approval number: GP_202107_010, 21 September 2021). All the participants received an information sheet that explained the purpose and procedure of the interview. The researcher obtained written informed consent from participants before the individual interviews and audio recordings. We assured all participants in writing of the study's confidentiality. The study maintained participant anonymity by not mentioning names; rather, it used alphabets to protect their identities. We also assured participants of their unrestricted right to withdraw from the study at any time. Although we procured the services of a clinical psychologist to manage the psychological concerns of the participants, the authors provided basic counselling. However, families who exhibited extreme anxiety and depression were referred to an experienced psychologist for professional treatment.

### 2.7 Rigour

The researchers (EKK, SS and ASA) ensured trustworthiness in qualitative research by applying the principles outlined by Ahmed and Amankwaa: credibility, confirmability, dependability and transferability [26, 27]. Credibility was enhanced through prolonged engagement, observations and the maintenance of an audit trail. Participants were engaged for approximately 30–40 min per interview, allowing for sufficient depth without overburdening them. An audit trail was maintained throughout the study, including documentation of interview summaries, coding decisions, theme development and analytical memos. All discussions and revisions during data analysis were logged to ensure transparency and confirmability. Multiple data sources, including semi‐structured interviews and detailed field notes, were used for triangulation. Member checking was conducted to strengthen credibility and confirmability. Following each interview, the researcher (SS) summarised the participants' responses and sought clarification to ensure their opinions were properly captured. Additionally, after transcription, transcripts were shared with participants to verify the accuracy of their accounts. Dependability was maintained by thoroughly documenting each research step and decision, ensuring traceability. Transferability was achieved by providing a detailed account of the study context, sample, sampling method and data collection process, allowing others to assess the applicability of findings to different settings.

### 2.8 Reflexivity

All researchers (EKK, SS and ASA) have doctorate degrees in nursing and are registered, trained nurses with extensive clinical experience caring for critically ill patients in the ICU. EKK and SS have worked in the study setting and cared for dying patients and their families in the ICU. All researchers have extensive experience in intensive care research and are aware of the ICU protocols and types of patients admitted to the adult ICU. We have a positive relationship with the ICU team and collaborate with them regularly on various research projects. While familiarity with the ICU context supported rapport‐building and contextual understanding, we acknowledge the potential insider bias. To mitigate this, researchers engaged in regular reflexive journaling, peer debriefing and discussions during the analysis to challenge assumptions and ensure interpretations remained grounded in participants' narratives rather than prior knowledge or expectations. The researchers' nursing training is comprehensive and includes psychiatric nursing; hence, they can provide basic counselling, recognise symptoms of distress and refer to an experienced psychologist for professional treatment when families exhibit extreme anxiety and depression.

## 3 Findings

A total of 7 participants were interviewed from an initial pool of 15 bereaved family members who were invited to participate in the study. Most participants were female, with a mean age of 45.2 years. All participants had a close relationship with the admitted patients until their death. The duration of patients' stays in the ICU prior to their death varied from 4 to 39 days (see Table 3).

**TABLE 3:** Participants characteristics.

ID	Gender	Age (years)	Relationship with patient	Patients' length of ICU stay
1	Female	57	Adult child	5 days
2	Female	43	Sibling	14 days
3	Female	61	Adult child	21 days
4	Female	72	Spouse	39 days
5	Male	31	Sibling	7 days
6	Male	69	Parent (father)	4 days
7	Female	74	Spouse	23 days

Three major themes and eight subthemes emerged after data analysis that described the experiences of bereaved family members. The main themes were emotional reactions and coping, desire for proximity and participation in care and communication and information sharing. The themes and subthemes are presented and supported by excerpts from the interviews as the qualitative findings. Refer to Table 4 for the details of themes, subthemes and codes from the data that supported the themes. The alphabet ‘F’ stands for family member. For example, F2 stands for family member participant number 2.

**TABLE 4:** Description of themes, subthemes and codes from family interviews.

Themes (3)	Subthemes (7)	Example codes
Theme 1: Emotional Reactions and Coping	Subtheme 1: Shock and numbness	It was like a shock Just standing For a moment I froze I felt numb and helpless
	Subtheme 2: Overwhelmed and emotional strain	It all happened so fast, he was gone We tried to be strong, but we cried inside' It was hard for me, my wife did not accept'
	Subtheme 3: Coping mechanisms	Spiritual beliefs help to cope Strong faith Prayers
Theme 2: Desire for proximity and participation in care	Subtheme 1: Need for physical presence	Just being there Sitting at the bedside Allowed to sit there
	Subtheme 2: Active involvement in care	Be able to change the bed Rub his hands Help with feeding Rub the feet with cream
	Subtheme 3: Spiritual support	Call the priest We are religious people Have the priest there
Theme 3: Communication and Information sharing	Subtheme 1: Importance of regular updates	Keep us informed Know what is happening Discuss options with the family
	Subtheme 2: Need for private communication	Visitors room Private space to share bad news It was not private

### 3.1 Theme 1: Emotional Reactions and Coping

The theme encompasses the emotional reactions from families during the treatment withdrawal and how they coped with the inevitable. The subthemes generated were ‘shock and numbness’, ‘overwhelmed and emotional strain’ and ‘coping mechanisms’.

#### 3.1.1 Shock and Numbness

Throughout the interviews, the bereaved family members appeared shocked upon receiving the news that their loved one's prognosis was poor, and treatment withdrawal was inevitable. The process happened so quickly for most families, and they could not cope with the situation.

I was frightened what did that mean… I felt totally numb and helpless…completely out of control and I did not know who to turn to help me. [F1]

…it was still a shock – I remember just standing there thinking but my brother is still there they have got it wrong. [F2]

I must admit for a moment I froze. I had never seen my father in that situation so it was a bit of a shock. My father did not look like my father. [F3]

It was like a shock. They did everything they could for our son. I think there was so much to think about what happens next… [F6]

#### 3.1.2 Overwhelmed and Emotional Strain

The treatment withdrawal process was overwhelming for most family members. It was emotionally draining and exhausting to accept the reality of losing a loved one. Some families expressed that

I think it all happened so fast he was gone in about 3 hours. I think it was a lot to take in. it was very emotional. I have never been in this situation before. [F2]

I think it was a lot to take in … we just sat there and tried to be strong but we were crying inside. [F8]

It was hard for me, it was even harder for my wife…my wife did not accept. She found it very difficult when we kept on telling her that it is true he is not going to make it and we must let him go. [F6]

#### 3.1.3 Coping Mechanisms

Despite the difficulties in accepting the reality of losing a loved one following treatment withdrawal, some families relied on their faith, prayer and religious beliefs to cope with the inevitable.

…our religious or spiritual beliefs help us to cope in these circumstances. [F3]

…She [participant's wife] has a strong faith, and she [participant's wife] prayed for our son to get better. When the doctor told us there was nothing more, they could do for our son… She thought he would get better because she prayed for him to get better. [F6]

…we knew we were religious people, and we knew whatever happens it will be His (pointing upwards to the sky) will. [F6]

### 3.2 Theme 2: Desire for Proximity and Participation in Care

Bereaved family members expressed their desire to be close to their loved ones and be able to participate in patient care. The theme consisted of three subthemes: ‘Need for physical presence’, ‘active involvement in care’ and ‘spiritual support’.

#### 3.2.1 Need for Physical Presence

Most family members preferred to be physically present for their relatives during the last stages of life. It was important for them to be close to their relatives, express their love and share the last moment with them. Two families had this to say

I would like to be near…I kept on thinking If only I could see my father if only I could hold him and tell him what was happening. [F1]

I think me being there – just sitting there was enough for my brother to know how much I loved him… [F2]

One other family member appreciated that their family was given enough time to be there for their loved one.

We were allowed to sit there at the bedside, we could talk to him and hold his hand and that was pretty much it. [F7]

#### 3.2.2 Active Involvement in Care

Some family members expressed their desire to be directly involved in the patient's care if they were allowed. After a decision was made to withdraw treatment, most families wanted to be around and be allowed to do simple and basic care such as bathing the patient, changing bed linen and applying cream on the patient's body. Two families recalled:

I can change a bed I can bath my father and rub cream on his skin. I can hold his hand and tell him how much we love him. I can sit there and tell him what is happening at home and why he must' not worry about those things that would normally worry him…I mean the family can do those things…. [F1]

Well, I can wash him, rub his hands and feet, talk to him or even read to him. I can do other things if the nurses show me how to do it…change the sheets or even turn him to rub his bum. [F2]

Family members appreciated being involved because it made them feel like they contributed to the care of their loved one at such a difficult time. One family member said

I noticed her skin was very dry so I asked if they would allow me to bring special cream for her. They did allow me to do that. So they allowed me to rub her full of cream after they had washed her. I enjoyed doing this because I felt it must make her feel relaxed and comfortable when her skin was not dry. I was very happy at doing the simple things like washing her, or brushing her, putting crème on her skin and her lips and even helping to feed her. [F3]

#### 3.2.3 Spiritual Support

Some family members relied on their religion as a supportive measure. However, they would have appreciated the inclusion of religious leaders and individuals while they went through the EOL process. They viewed these as effective steps to provide spiritual support for the grieving families.

…we knew we were religious people and we knew whatever happens it will be His (pointing upwards to the sky) will. [F6]

…having a priest there would also be helpful. If our Priest had been called he would have come to be there with me to hear the sad news and also to support me. [F5]

### 3.3 Theme 3: Communication and Information Sharing

Communication and information sharing were another important theme that emerged from the data. Two subthemes were generated: ‘Importance of regular updates’ and ‘need for private communication’.

#### 3.3.1 Importance of Regular Updates

Some family members recalled the regular updates they received on patients' treatments, even until the end. They emphasised that the healthcare team provided them with frequent information on the patient's prognosis, treatments given, and any changes that occurred at every point in the patient's care. They appreciated the access to information and support provided by attending nurses and doctors, as shown in the quotes below:

We knew everything exactly what was happening all the time. They told us about the treatment all the time and how he was holding up. They even told us what the blood results were and when they were changing the treatments and starting new things. [F2]

Yes, they kept us informed all the way. I felt we had a good understanding of when treatments were happening and what the effects were. I found the doctors were always available to discuss options with you. I also found the nursing staff were very kind and supportive when they were caring for my sister [F5]

#### 3.3.2 Need for Private Communication

Some families also highlighted the need for privacy during the dissemination of sensitive information on the patient's prognosis. They expressed that openly discussing a patient's prognosis in the presence of other patients' families and visitors was inappropriate and compromised patient and family privacy. One family member said

I found the doctors came to speak to me in the visitor's room when all the other family members were there. It was not private and it also could make the other people anxious or worry about what was going to happen to their patients [F6]

Another family member emphasised the need to have a special private room within the hospital to share such delicate information with patients' families. In such a place, they are able express their emotions fully. One family member expressed her thoughts as follows:

…it would be nice for families to have a special place where they can sit and wait to hear what is happening to the patient. I think a private room would be better to share bad news with family. Yes, then you can express your emotions [F5]

## 4 Discussion

The study explored bereaved family members' experiences of treatment withdrawal and end‐of‐life care in the adult ICU. Three main themes emerged: emotional reactions and coping, desire for proximity and participation in care, and communication and information sharing. Bereaved families experienced grief following treatment withdrawal and relied on personal rituals, faith, and social support as they dealt with the intense emotions, including distress and sadness. They looked for spiritual support, active participation, and physical presence. Compassionate, transparent communication and emotional support greatly influenced their experience and perception of care. The findings offer a broad overview of the withdrawal of life‐sustaining treatment from the perspective of a bereaved family. Healthcare providers in ICUs may use these findings to fully understand the grief symptoms patients' families experience during treatment withdrawal and implement appropriate family‐centred care approaches to support these families.

This study highlights that bereaved families experience a multifaceted and highly personalised range of grief responses, with anticipatory grief being particularly prominent throughout the dying process. Immediate reactions such as shock and sadness often emerged not only in response to the patient's death but also as part of an ongoing process that begins prior to the occurrence of death. Grief, encompassing anticipatory and post‐bereavement phases, is a complex and deeply personal experience frequently encountered by families in the ICU, particularly during treatment withdrawal and EOL care [2, 28, 29]. Consistent with previous research, families often report a range of intense emotions upon learning of the impending transition to EOL care and during bereavement [1, 2, 30]. These multifaceted responses reflect not only the weight of the impending loss but also the relational disruption and psychological uncertainty that accompany it. The nature and intensity of these grief responses may be influenced by various factors, including the circumstances surrounding the patient's death, caregiver experiences, social support, family coping strategies, communication quality, advance care planning, limited involvement in patient care and the availability of post‐loss support systems [28, 29, 31]. Hence, poor communication and insufficient psychosocial support can intensify grief, whereas structured EOL family conferences and counselling have been shown to help manage expectations, provide emotional support and assist families in navigating their grief effectively [32, 33, 34]. These findings underscore the importance of high‐quality proactive communication and the integration of family‐centred care practices, including involving families in decision‐making and offering continuous support throughout the ICU stay.

Grief can have a profound emotional, relational and enduring impact on bereaved families [2, 28, 35]. Beyond the immediate emotional impact, grief disrupts established family roles and personal identities, requiring individuals to adapt to new responsibilities and dynamics within the family unit [36, 37]. When grief becomes intense or prolonged, it may evolve into prolonged grief disorder, further impairing a family's ability to return to everyday functioning [31, 35]. These consequences highlight the importance of providing timely and appropriate support to help families navigate the uncertainty and emotional distress associated with EOL experiences. A range of supportive interventions has been identified as helpful in assisting families during this period, including family conferences, bereavement follow‐ups, condolence letters, ICU diaries, follow‐up phone calls, counselling services and peer support groups [33, 38, 39]. Findings from the current study revealed that some bereaved families drew strength from spiritual and religious beliefs, using faith and prayers as immediate coping mechanisms to make sense of their loss. For many, these practices were preferred over formal psychological or bereavement interventions. While spiritual coping may provide comfort and meaning, it may not fully address the complex psychological and emotional dimensions of grief. Without comprehensive support, families remain vulnerable to adverse mental health outcomes, including prolonged grief, depression, and PTSD [31, 35].

Bereaved families often experience substantial unmet emotional and bereavement care needs, both before and after a patient's death in the ICU [40, 41]. This highlights the need to prioritise the emotional and psychological well‐being of families by implementing bereavement support interventions. Such measures may include grief counselling, support workshops, grief informational booklets, condolence letters and follow‐up meetings [33, 38, 39]. These strategies have demonstrated effectiveness in reducing complicated grief, PTSD and depression in well‐resourced and adequately staffed ICU settings. However, implementing bereavement programmes in resource‐limited settings, such as many South African ICUs, remains challenging due to constrained infrastructure and workforce shortages. Additionally, cultural and societal perceptions of death, including how and when it occurs, can profoundly influence experiences of treatment withdrawal, EOL decision‐making and bereavement care. These contextual factors underscore the need for targeted staff training in bereavement care and communication skills to ensure culturally sensitive and compassionate care. Early integration of palliative care and psychological support may also assist families in navigating grief during the transition to EOL care. However, in many South African ICU settings, access to such palliative care services is often limited. Future intervention studies could explore context‐appropriate models for integrating palliative care into ICU practice to enhance bereavement support and family outcomes.

This study reinforces a growing body of evidence indicating that family presence, involvement, and preparedness during and after treatment withdrawals in the ICU are essential for improving EOL care experiences and bereavement outcomes. Families who receive adequate support through structured communication, shared decision‐making, involvement in patient care and continuous bedside presence report greater satisfaction with care, feel more prepared for the patient's death and experience fewer symptoms of PTSD, depression and prolonged grief [28, 30, 32, 42, 43]. These findings are echoed in the present study, where participants consistently emphasised the value of being present and actively engaged, particularly during and after treatment withdrawals. In contrast, families who were unable to say goodbye or participate meaningfully in EOL decisions described heightened emotional distress, contributing to more complicated grief responses [29]. The degree to which families are able to be present and involved largely depends on the preparation and support they receive throughout the dying process. Early engagement and clear, compassionate communication about what to expect can significantly ease this transition and mitigate psychological distress [44, 45].

Our findings support calls for more flexible visiting policies during EOL care to allow sustained bedside presence and meaningful final interactions. While concerns about operational efficiency and infection control persist, open visitation has been shown to improve family coping, foster emotional closure and reduce symptoms of anxiety and depression after a loved one's death [46, 47]. Therefore, a shift towards a more inclusive, family‐centred model of ICU care is warranted. The model should not only cater to the dying patient's needs but also to the emotional and psychological health of those who remain behind. Achieving such an objective will require re‐evaluation of individual institutional visitation policies, with clearly defined guidelines and input from key stakeholders to ensure that families receive consistent, compassionate support during some of the most vulnerable moments of their lives.

This research demonstrates the critical role of effective communication in supporting bereaved families during treatment withdrawal and following the death of a loved one in the ICU. Previous studies have shown that when families are inadequately informed about the dying process and the implications of treatment withdrawal, anticipatory grief may be intensified and emotional preparedness compromised [9, 17, 32]. Conversely, families who receive timely and accurate information about what to expect are better prepared for the patient's death and report greater emotional readiness [42, 43]. Clear, compassionate and culturally sensitive communication, coupled with shared decision‐making, is essential for meeting families' EOL needs and preparing them for the events ahead [9, 17, 48, 49]. Consistent and transparent information sharing by healthcare providers has been shown to ease emotional distress, reduce anxiety and depressive symptoms and promote a healthier grieving process [28, 45]. Our findings align with this evidence, revealing that effective communication enhances family satisfaction with EOL care and supports emotional adjustments during bereavement. In the South African context, cultural and religious beliefs significantly shape how families interpret treatment withdrawal and EOL care. The region's ethnic and language diversity further illustrates the importance of culturally sensitive communication strategies. Healthcare providers must engage in linguistically accessible and culturally sensitive communication to ensure that all families receive appropriate and respectful support. This approach not only fosters trust between healthcare providers and families but also encourages open discussions about preferences and expectations regarding end‐of‐life care. By prioritising these communication strategies, the healthcare system can better meet the unique needs of diverse communities, ultimately leading to improved quality care and support during challenging times.

Most families in this study expressed a clear preference for receiving sensitive EOL information in private settings, illustrating the value of confidentiality and dignity during periods of clinical uncertainty. However, the design of most ICUs, characterised by multi‐bedrooms and shared waiting areas, was identified as a significant barrier to maintaining privacy [50, 51]. These environmental constraints often limit healthcare providers' ability to engage in confidential, compassionate conversations with families, particularly during emotionally charged moments of care planning or treatment withdrawal [51, 52]. These insights point to a systemic need for designated private spaces within ICU environments where sensitive discussions can be conducted, and families receive emotional support in a more respectful and secure setting. Proactively initiating private conversations early is essential, especially when anticipating a transition to EOL care, to ensure that families feel supported, respected and well‐informed throughout the dying process. Such environments not only facilitate open dialogue but also help in building trust between healthcare providers and families.

Bedside nurses assume a critical role in supporting families through presence, emotional support and clarification of clinical information, which could help families navigate the grieving process. As frontline providers of patient and family care, they are uniquely positioned to bridge the limited emotional and psychological support, communication challenges, systemic constraints and bereavement support that many families experience during and after EOL in the ICU, especially in resource‐constrained settings like South Africa [14, 15, 20]. Nurses can identify signs of distress, initiate conversations about prognosis and treatment withdrawal and serve as liaisons between physicians and families. They are also well positioned to support culturally and spiritually appropriate rituals, provide presence during the dying process and advocate for compassionate communication [6, 20, 32]. Training nurses in bereavement care, grief support, family communication skills and cultural competence is critical to strengthening the support offered to families. Even in settings where formal bereavement services are unavailable, nurses' engagement can ensure the recognition and addressing of families' emotional needs. The study proposes the adoption of a holistic, family‐centred model of care in ICUs, including those in South Africa, to better support the emotional, spiritual and practical needs of families before, during and after the death of a loved one. This approach not only fosters a compassionate environment but also empowers families to navigate their grief more effectively.

### 4.1 Study Limitations

The findings may help improve end‐of‐life care for patients and families, but the study has limitations. It was conducted in a single 18‐bed multidisciplinary ICU at an academic referral hospital, limiting generalisability. Future studies should include other adult ICUs in the hospital. Recruitment was challenging due to COVID‐19 restrictions, ICU visiting protocols and the emotional impact of loss. Despite the limited ICU access, many families were able to participate. Data were collected 6 months or more post‐bereavement, presenting challenges due to some participants still experiencing grief, while others had relocated or were unavailable. The 6‐month bereavement period also presented some potential for recall bias, another limitation of the study. The small and relatively homogenous sample is also acknowledged as a limitation of the study. Despite these challenges, the study offers a foundation for future research in this area.

### 4.2 Implications for Practice

The study emphasises the need for family‐centred end‐of‐life (EOL) care in ICUs. It suggests ways to improve communication, encourage family involvement, provide emotional and psychological support, integrate spiritual care and redesign the ICU layout for privacy. Effective communication is essential for families to become well informed and emotionally prepared for treatment withdrawal. Open visitation and standardised referrals to psychologists, palliative care teams and social workers can help families cope with their grief. Integrating spiritual care into end‐of‐life practices can improve family satisfaction. Reevaluating the ICU layout and design for privacy can enhance both privacy and emotional support. To improve the quality of EOL care, incorporating EOL care education into nursing and medical curricula is proposed.

## 5 Conclusion

Bereaved family members' experiences of treatment withdrawal and EOL care in the ICU are complex and multifaceted, shaped by emotional, spiritual and communication dynamics. Many families are unprepared for the inevitable outcomes and have diverse needs, including information, emotional support, spiritual support, proximity and patient comfort. Supporting families during and after treatment withdrawal is essential. Effective communication, family involvement and interdisciplinary teamwork are crucial for quality end‐of‐life care. Healthcare providers should view the patient and family as a unit of care, ensuring clear communication and emotional support while creating a compassionate environment to help families navigate treatment withdrawal and the grieving process. To address this gap, there is a need to incorporate family‐centred care principles into end‐of‐life (EOL) care in the ICUs to maximise the participation of families in EOL care. Future research could validate our findings by expanding the study population to include nurses and physicians.

## Ethics Statement

Ethics approval was obtained from the Human Research Ethics Committee (Medical) of the University of the Witwatersrand (Protocol Number: M210229, 17 September 2021) and the Johannesburg Academic Hospital (Ethic approval number: GP_202107_010, 21 September 2021).

## Consent

Before beginning study procedures, participants signed an informed consent form, a data privacy agreement and consented to the audio‐recording of the interview.

## Conflicts of Interest

The authors declare no conflicts of interest.

## Data Availability

The data that support the findings of this study are available on request from the corresponding author. The data are not publicly available due to privacy or ethical restrictions.
